# Cultural Transmission, Evolution, and Revolution in Vocal Displays: Insights From Bird and Whale Song

**DOI:** 10.3389/fpsyg.2020.544929

**Published:** 2020-09-29

**Authors:** Ellen C. Garland, Peter K. McGregor

**Affiliations:** ^1^Centre for Social Learning and Cognitive Evolution, and Sea Mammal Research Unit, School of Biology, University of St. Andrews, St. Andrews, United Kingdom; ^2^Eco-Ethology Research Unit, ISPA-Instituto Universitário, Lisbon, Portugal

**Keywords:** birdsong, whale song, vocal learning, cultural transmission, cultural evolution, cultural revolution, local dialect, sexual selection

## Abstract

Culture, defined as shared behavior or information within a community acquired through some form of social learning from conspecifics, is now suggested to act as a second inheritance system. Cultural processes are important in a wide variety of vertebrate species. Birdsong provides a classic example of cultural processes: cultural transmission, where changes in a shared song are learned from surrounding conspecifics, and cultural evolution, where the patterns of songs change through time. This form of cultural transmission of information has features that are different in speed and form from genetic transmission. More recently, culture, vocal traditions, and an extreme form of song evolution have been documented in cetaceans. Humpback whale song “revolutions,” where the single population-wide shared song type is rapidly replaced by a new, novel song type introduced from a neighboring population, represents an extraordinary example of ocean basin-wide cultural transmission rivaled in its geographic extent only by humans. In this review, we examine the cultural evolutions and revolutions present in some birdsong and whale song, respectively. By taking a comparative approach to these cultural processes, we review the existing evidence to understand the similarities and differences for their patterns of expression and the underlying drivers, including anthropogenic influences, which may shape them. Finally, we encourage future studies to explore the role of innovation vs. production errors in song evolution, the fitness information present in song, and how human-induced changes in population sizes, trajectories, and migratory connections facilitating cultural transmission may be driving song revolutions.

## Introduction

Some of the most important aspects of animals’ lives have a large learned component (Whiten et al., 2017). Learning plays an important role in, for example, tool use (Whiten et al., 1999), foraging specializations (Allen et al., 2013; Aplin et al., 2015), and migratory routes (Carroll et al., 2015). Vocal communication provides another good example: many species of birds socially learn details of the songs that underpin successful resource defense and breeding, while a few whale species show signs of song learning that likely contributes to their reproductive success. This form of cultural transmission of information has features that are different in speed and form from genetic transmission (see Aplin, 2019, for a recent review), and these features have given cultural transmission a role in one of the major transitions in life (*sensu* Maynard Smith and Szathmary, 1999). Studies of birdsong have provided a wealth of information on learning mechanisms, including social learning aspects and the underlying hormonal and neural pathways, and these have contributed to our understanding of cultural evolution in general. Production learning is a form of social learning, where as a result of experience with signals of other individuals, an animal learns to modify the form of its own signal (Janik and Slater, 2000; Naguib et al., 2009; Janik, 2014). Production learning is considered rare in vertebrates. As whales and songbirds are thought of as exemplars of this advanced form of social learning, it is reasonable to adopt a comparative approach to better understand the factors driving their evolution. For example, the pattern of song “revolutions” in humpback whales (*Megaptera novaeangliae*), where the single population-wide shared song type is rapidly replaced by a new, novel song type introduced from a neighboring population, is unique in whales (Noad et al., 2000; Garland et al., 2011). The rapid (within season) and extensive (ocean basin-wide) spreading waves of song replacement represents an extreme example of song change that has the potential to increase our understanding of cultural transmission in general. While cultural evolution of song in birds has been widely documented (e.g., Williams et al., 2013; Pipek et al., 2018), sometimes at a continent-wide scale (e.g., Otter et al., 2020), we are aware of only one songbird species, the corn bunting [*Emberiza (Miliaria) calandra*], that shows concerted change in details of local dialects from year to year (McGregor et al., 1997). Therefore, the aim of this paper is to compare humpback whale song revolutions with the cultural evolution of song in birds (focusing on corn buntings) and to identify the sort of data that would be required to test some of the possible explanations, both in birds and whales.

Controlled-environment studies of factors influencing whale song cultural transmission in both field and lab experiments are unlikely in the foreseeable future because of insurmountable practicalities; for example, humpback whales are 14 m long, weigh 30 tonnes, and migrate ~6,000 km one-way. While agent-based models can provide an informative framework for investigating various social learning scenarios (Mcloughlin et al., 2018), all types of model can lack real-world applicability. Therefore, a comparative approach is currently the best option to understand the key factors of such phenomena. Information from lab studies of song learning in birds may seem the obvious comparison; however, such studies are problematic to interpret and apply to a real-world setting because virtually all such studies lack relevant social context. This is a significant limitation of lab studies because a social context is a prerequisite for song learning in nature and because song-learning mechanisms have evolved in a social environment. Cultural transmission of birdsong has been studied in the field, with individually identifiable subjects followed for some generations, for several species. Examples include the pioneering study on song sparrows *Melospiza melodia* (Nice, 1943; since extended by Beecher’s lab, e.g., Beecher, 2017; Beecher et al., 2020), Bewick’s wren *Thryothorus bewickii* (Kroodsma, 1974), saddlebacks *Philesturnus carunculatus* (Jenkins, 1978) and *Philesturnus rufusater* (Parker et al., 2012), indigo buntings *Passerina cyanea* (Payne, 1981; Payne and Payne, 1993), great tits *Parus major* (McGregor and Krebs, 1982), and savannah sparrows *Passerculus sandwichiensis* (Wheelwright et al., 2008). An approach using unidentified singers recorded continent‐ or country-wide by many workers including citizen scientists (e.g., Pipek et al., 2018; Otter et al., 2020) can allow cultural evolution to be documented on comparable spatial scales to whale song. While lacking the control of lab studies, field studies do take place in a natural social context. Field studies have produced sufficient indications of patterns and factors affecting song learning in birds to be useful in a comparative approach to understanding humpback song revolutions.

## Whale Song: From Simple Songs to Cultural Revolutions

**Figure 1:**
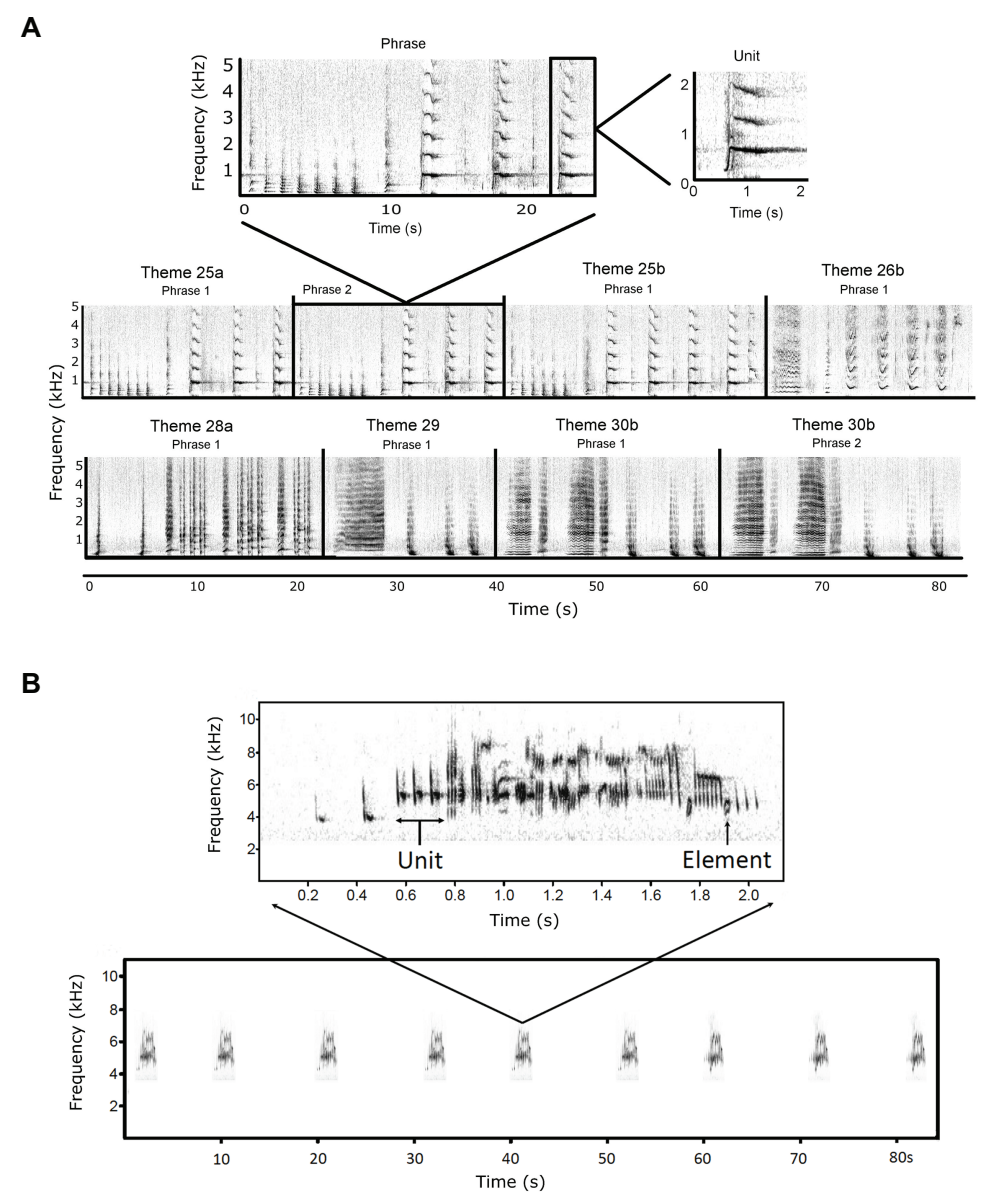
**(A)** Spectrograms illustrating the hierarchical structure of humpback whale song (from Garland et al., 2017b). A single unit (“trumpet”) and a single phrase from Theme 25a are shown in the top panel. Theme 25a units from the single phrase in the top panel are as follows: short ascending moan, grunt, grunt, grunt, grunt, grunt, grunt, short ascending moan, trumpet, squeak, trumpet, squeak, and trumpet. The repetition of phrases and the sequential singing of themes are shown in each of the subsequent panels. Spectrograms were 2048 point fast Fourier transform (FFT), Hann window, 31 Hz resolution, and 75% overlap, generated in Raven Pro 1.4. Reprinted with permission from Garland et al. 2017b (Copyright 2017, Acoustic Society of America). **(B)** Spectrograms illustrating the hierarchical structure of corn bunting song. The lower panel represents part of a bout of singing lasting several minutes. It shows two song types (song types differ mainly in the first part of the song; McGregor, 1986) sung in an eventual variety style: five repeats of song type 1, then four repeats of song type 2. A single song (top panel) is composed of units of grouped elements, where elements are single continuous sounds. Spectrograms produced with 20 kHz sampling rate and 160 Hz bandwidth.

Most baleen whale species sing. Like songbirds, their songs range from simple songs comprised of a few sound types (e.g., fin whales, *Balaenoptera physalus*: Delarue et al., 2009 and blue whales, *Balaenoptera musculus*: Stafford et al., 2001) through the complex songs of bowhead (*Balaena mysticetus*; Stafford et al., 2018) and humpback whales (Payne and McVay, 1971). While cultural transmission of vocalizations is not limited to song in cetaceans (e.g., killer whale and sperm whale repertoires; Ford, 1991; Rendell and Whitehead, 2003), the aim of this review is to examine the cultural transmission of male song displays. To that end, male humpback whales sing a long, complex, stereotyped, and hierarchically structured vocal sexual display termed “song” (Payne and McVay, 1971; Herman and Tavolga, 1980). It is generally agreed that song functions in sexual selection, but the details of whether its primary function is to attract a mate, mediate male–male interactions, or as a multi-message signal, are currently contested (see review by Herman, 2017). In terms of the comparative approach taken in this paper, both humpback whales and corn buntings are producing complex patterns that change over time; we compare the ways in which these patterns change. A humpback whale song *unit* is considered analogous to a birdsong *note* or *element*, and a humpback whale song *phrase* (itself consisting of a stereotyped sequence of units; Payne and McVay, 1971) is considered analogous to a bird’s *song* (Figure 1; Cholewiak et al., 2013). In the humpback whale song hierarchy, phrases are repeated multiple times to make a *theme*, and a sequence of different themes forms the *song* (Payne and McVay, 1971).

A single humpback whale song can last from 5 to 30 min, and a song session can last for many hours (Payne and McVay, 1971). All humpback whales produce units with predictable acoustic characteristics that are similar within and across whales and populations, making it possible to compare songs. From this, we hypothesize that there may be a general, worldwide repertoire of unit types (although the exact number of unit types per location is a current focus of research), suggesting that humpbacks may possess an inherited basic template of own species sound units. It is the arrangement and rearrangement of units into distinct, stereotyped patterns (like with birdsong) that is the major focus of humpback song research. Humpback whales are considered “*eventual variety*” singers (Cholewiak et al., 2013, a term that originated in birdsong; Hartshorne, 1956), as they repeat phrases within each theme before moving onto the next theme (e.g., AAAA, BBB, CCC, etc.). At any point in time, most males within a population will sing the same song type, thus, there is strong cultural conformity to the current arrangement (version) of the song display (Payne et al., 1983; Payne and Payne, 1985).

Song evolution occurs in humpback whale song through addition, deletion, and/or substitution at all levels within the nested song hierarchy. Individual sound units can be stretched and split into 2 units, new themes can be added, and older themes deleted from the song (Winn and Winn, 1978; Payne et al., 1983). The process is one-way; no song has reappeared once it has been dropped entirely by a population (Winn and Winn, 1978; Payne and Payne, 1985; Cato, 1991). As the humpback song changes, all males within the population incorporate the changes into their own song, maintaining the observed cultural conformity. A salient feature of humpback song is strong cultural conformity; they may learn the changes to the song from surrounding males in acoustic contact or innovate their own changes. Recent work into song learning suggests that humpback whales use learning mechanisms similar to those of songbirds and humans when acquiring new material (Garland et al., 2017a). The song is segmented into themes and learned as separate segments. New themes can evolve from older themes or can be completely new arrangements of units (Payne and Payne, 1985) created *de novo*. However, which male(s) innovate or create new *de novo* arrangements and who ultimately leads the evolutionary song progression remains elusive.

At the ocean basin scale, songs are similar (based on “matched” themes/phrase types), but this similarity depends on the geographical distance and variable interactions among populations (Payne and Guinee, 1983; Helweg et al., 1998; Darling and Sousa-Lima, 2005; Darling et al., 2019). Song sharing among populations is hypothesized to occur due to three mechanisms (Payne and Guinee, 1983): song sharing on shared feeding grounds and/or on shared or partially shared migratory routes, through males visiting more than one wintering ground in consecutive years, and by males visiting more than one wintering ground within a breeding season (McSweeney et al., 1989; Clapham and Mattila, 1990; Calambokidis et al., 2001; Clark and Clapham, 2004; Smith, 2009; Garrigue et al., 2011; Stimpert et al., 2012; Garland et al., 2013a; Owen et al., 2019). A recent study by Darling et al. (2019) in the North Pacific demonstrated a single song type with varying amounts of theme sharing among the four North Pacific populations, mirroring previous studies (Helweg et al., 1990, 1992; Darling et al., 2014). There were year-to-year regional differences among populations suggesting variable interactions, as geographic distance was not a predictor (but was a factor) in song similarity (Darling et al., 2019). The authors suggest that differences among these populations are best viewed as “fluid divergences from a more common [single] North Pacific song.” In contrast, in 2000, Noad et al. (2000) documented an extraordinary cultural phenomenon: the song from the west Australian population of humpback whales appeared in the east Australian population and rapidly replaced the existing song. This process was termed a “*song revolution*” to clearly distinguish it from the common and much slower process of song evolution as described above (Noad et al., 2000). The complete replacement of a population-wide display was striking; Noad et al. (2000) were able to trace this process over 2 years where the new song first appeared in low numbers and then increased in frequency until the old song was completely gone. The movement of a small number of whales from the west Australian population to the east Australian population taking their song with them was hypothesized to have initiated the cultural transmission of the song between the ocean basins. Most importantly, this cultural phenomenon (song revolution) was believed to be a unique event.

**Figure 2:**
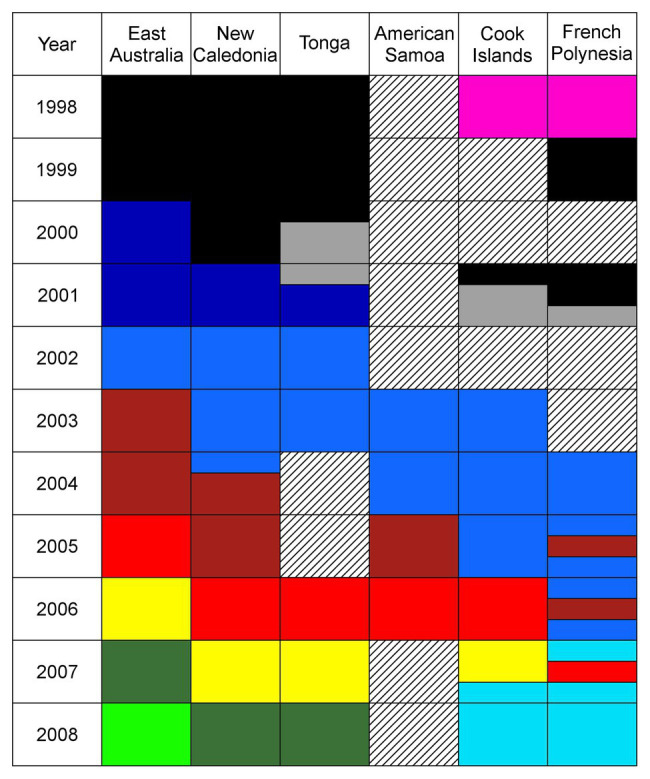
Humpback whale song types identified in the South Pacific region from 1998 to 2008 (from Garland et al., 2011). Populations are listed from west to east across the region. Each color represents a distinct song type; song type colors are as follows: black, gray, pink, dark blue, blue, light blue, dark red, light red, yellow, dark green, and light green. Two colors within the same year and location indicate that both song types were present. In these cases, the seasons are broken into three periods (early, middle, and late) to indicate when a new song type was recorded. Crosshatching indicates no data available. Reproduced with permission.

This was however not the case. Garland et al. (2011, 2012, 2013a,b, 2015, 2017a) have conclusively demonstrated multiple song revolutions where multiple song types from the east Australian population have been horizontally transmitted east across the populations in the South Pacific in a series of cultural waves spanning a decade (Figure 2). Song types take 2 years to spread from east Australia across to French Polynesia in a series of steps creating a checkerboard of behavioral phenotypes at the decadal scale. Recently, a variant of white-throated sparrow (*Zonotrichia albicolis*) song spread across Canada in a west to east direction replacing the existing song; the spread was similar in the extent of change, but much slower in that the change took some decades (Otter et al., 2020). Humpback whale song dynamics in the South Pacific appear to differ from other ocean basins as several different song types can be present at any point in time instead of a single song type (Darling et al., 2019). The unidirectional cultural transmission of song types is hypothesized to occur due to differences in population sizes across the region; novel song types appear to spread from larger to smaller populations (Garland et al., 2011, 2015). Rapid and complete replacement of a behavioral phenotype is a striking cultural process. The only mechanism to date that appears to trigger a song revolution is the appearance in an ocean basin of a new song type that can be traced to come from another (i.e., song from the west Australian population located in the Indian Ocean appearing in the east Australian population in the South Pacific). We speculate this phenomenon occurs throughout the Southern Hemisphere due to humpbacks’ circumpolar feeding grounds and no landmasses to impede movement at high latitude. We would not expect to find such revolutionary dynamics in Northern Hemisphere populations (primarily located in the North Pacific and North Atlantic) because these populations are constrained by continents on each side of the ocean basins. However, there may be the possibility of trans-hemisphere cultural transmission of song (e.g., North and South Pacific breeding grounds may overlap along the northern South American and central American coasts). What is clear, though, is that when a novel song appears within a population, singers switch to the novel song. Novel songs are hypothesized to be rapidly learned, and this quest for novelty is thought to be driven by sexual selection (Noad et al., 2000; Cerchio et al., 2001). This would allow a male to stand out against the background of song. However, this novelty needs to be constrained; otherwise, all songs would rapidly diverge at the individual level (Noad, 2002), which is not what we observe.

Understanding the underlying mechanisms of this process and the individual learning strategies that create such a pattern is challenging. Recently, Garland et al. (2017a,b) investigated how humpback whales learn a new song type by examining instances of song hybridization, where we recorded a whale hypothesized to be in the process of learning a new song. Songs were segmented and then learned as whole themes, akin to how songbirds learn song and human infants learn language in segments (Garland et al., 2017a). The position in the song a singer switched from “old” to “new” song themes was not random; a “*switch when similar rule*” was uncovered where singers smoothly transitioned from old to new themes where the similarity in unit type and arrangement was highest (Garland et al., 2017a). Agent-based models of humpback whale song revolutions have also provided valuable information on the various scenarios that may initiate a song revolution (Lamoni, 2018). By modeling the west–east Australian song revolutions and comparing this to actual song revolution data, individual agents required a song memory to ensure they did not immediately revert to their old song. This hints at a cognitive capacity to remember not only the current song but also the previous season’s song, similar to songbirds’ discrimination learning (e.g., McGregor and Avery, 1986).

The appearance of a new song type presents the whales with a substantial amount of novel material that requires rapid learning to mirror the pattern we observe. Allen et al. (2018) investigated the complexity of song in the east Australian population over 13 consecutive years. As songs evolved, complexity increased; however, song revolutions were significantly less complex than their predecessor. The relative complexity of songs in song revolutions may represent an upper limit to song learning (Allen et al., 2018). It may also support the “cognitive capacity hypothesis,” where song complexity may convey an individual’s overall cognitive ability and could be used by prospective mates. Finally, this cyclic pattern of increasing complexity during evolutions and decrease in complexity with a novel, revolutionary song may superficially mirror broad-scale human music evolutionary processes (e.g., Baroque to Classical and then into the Romantic period).

## Song Learning in Birds

The general pattern of song learning in songbirds is commonly described as an inherited basic template of own species song and a predisposition to add details from singing adults (often fathers) within a sensitive period in early life (when nestling or fledgling), after which the song is unchanging (e.g., Brainard and Doupe, 2002). This pattern is apparent in lab studies of song learning using tape tutoring (see Mennill et al., 2018, for tape tutoring in the field), i.e., in the absence of social interaction. The potential importance of male–male interaction in driving song learning was shown by a lab experiment using live tutors (Baptista and Petrinovitch, 1984) – males learned the song of their social tutor even when it was a different species. The importance of social interaction in song acquisition has been shown in several other bird species (e.g., Farabaugh et al., 1994; Nordby et al., 2000; Poirier et al., 2004; Beecher, 2017), discussed in relation to the “classic” song-learning pattern (e.g., Beecher and Burt, 2004; Beecher and Brenowitz, 2005) and linked to human speech acquisition (e.g., Kuhl, 2007). Given that male–male interaction, including singing interactions, is a prerequisite for establishing a territory in songbirds, there is good reason to expect natural song learning to be affected by such interactions. Indeed, field studies of juvenile song sparrows show they are particularly attracted to adult singing interactions during their prime song-learning phase (Templeton et al., 2010). Below, we explore two patterns of song learning in the field that may be salient in understanding song learning in humpback whales.

### Field Evidence for Song Learning: A Clinal Pattern, e.g., Great Tits

**Figure 3:**
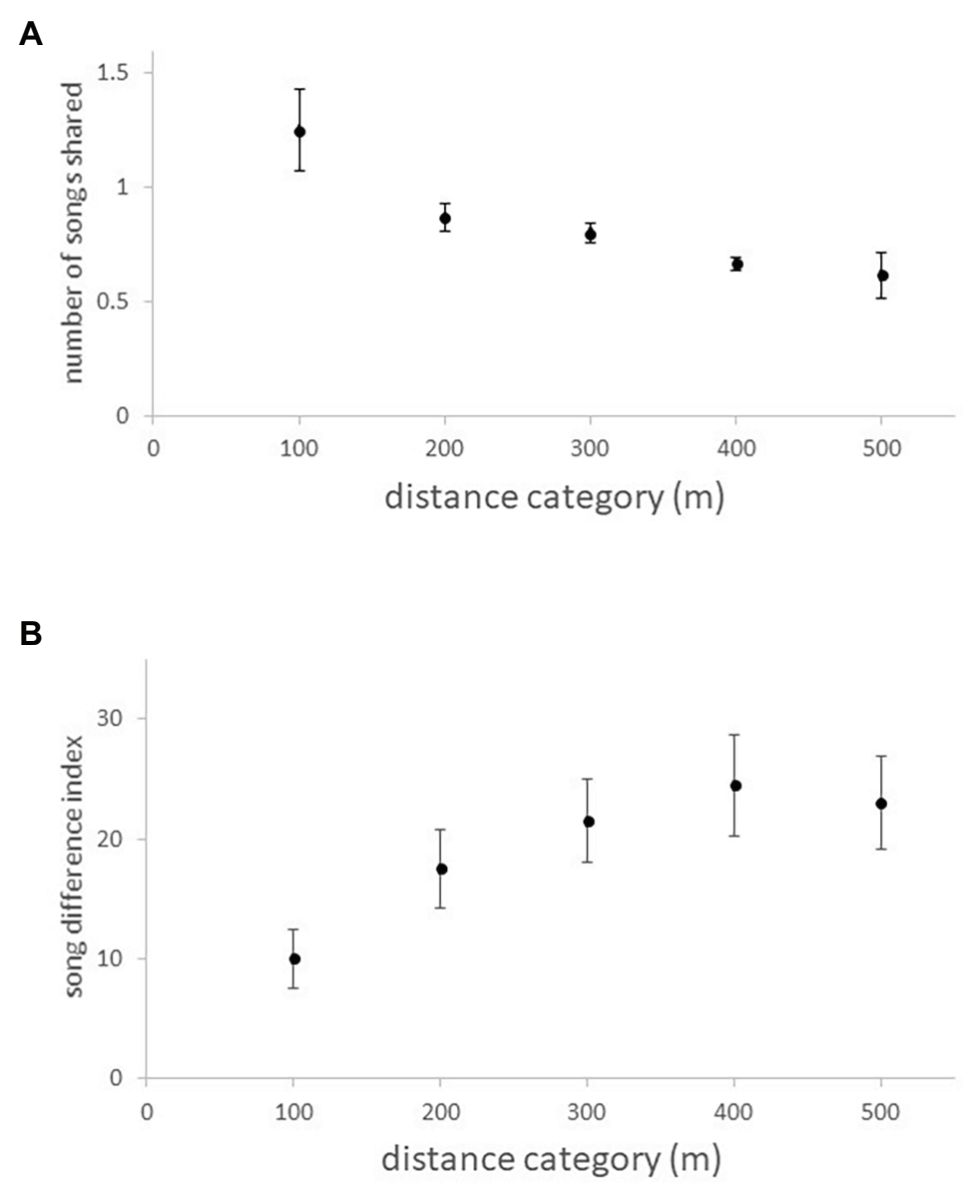
Changes in song similarity (values are means ± SE) with distance between pairs of male great tits in five distance categories (100 m increments, 100 m ≈ 1 territory width). **(A)** The extent of repertoire sharing, expressed as the difference from the average yearly rate. *n* = 30 (five distance categories for each of 6 years). Details in McGregor and Krebs (1982). **(B)** The dissimilarity between songs of the same song type, expressed as a difference index (details in Falls et al., 1982). *n* = 150 comparisons.

Male great tits defend a territory in the breeding season and pair with a (non-singing) female. The role of song in territory defense is well-established (Krebs et al., 1978). Males sing a repertoire of one to six discrete song types (McGregor and Krebs, 1982) and are known to perceive song type categories in a similar way to researchers’ visual categorization of spectrograms (e.g., Falls et al., 1982; Shy et al., 1986; Weary, 1990). Evidence that song is learned mainly from territorial neighbors is two-fold: the extent of repertoire sharing with a focal male declines with increasing distance from the male (Figure 3A), as does the similarity of details of songs of the same song type (Figure 3B; Falls et al., 1982), and the details of song types shared between sons and fathers are less similar than song types shared between sons and males in the sons’ year of breeding (McGregor and Krebs, 1982). The advantage of sharing song types with neighbors is likely related to song type matching (e.g., Falls et al., 1982; King and McGregor, 2016) in aggressive singing interactions between males. Field studies of individually marked males have shown that song learning can continue throughout life [for both production (McGregor and Krebs, 1989) and discrimination (McGregor and Avery, 1986) learning], with the stimulus for learning being interactions with male neighbors. Interestingly, it seems that songs learned for discrimination are not forgotten and create proactive memory interference (McGregor and Avery, 1986), whereas songs for production are added and others not used with the effect that repertoire size does not change significantly with age (McGregor et al., 1981; McGregor and Krebs, 1989). The result of this pattern of song learning is that song types change in commonness in the population across years mainly due to males leaving/arriving (McGregor and Krebs, 1989) and that both repertoire and song type similarity decline with distance from a focal male. Similar patterns are seen in other territorial, discontinuous-singing, repertoire species (examples in Hill et al., 1999).

### Field Evidence for Song Learning: Local Dialects, e.g., Corn Bunting

In contrast to the gradual, clinal change in song similarity described in the previous section, other species show a mosaic pattern of song similarity, with high similarity within a local dialect and abrupt changes in similarity at boundaries between contiguous local dialects. Such a pattern is often termed microgeographic variation to acknowledge that the distances between local dialects are too small to create the differences by geographical isolation. The corn bunting shows clear local dialect variation in song in the United Kingdom (McGregor, 1980; Holland et al., 1996), France (Pellerin, 1981), Poland (Osiejuk and Ratynska, 2003), and Portugal (Latruffe et al., 2000). Within an area of suitable habitat, the population is fragmented into a number of local dialects, song variation is discrete, and no examples of intermediate or mixed songs have been found, although some males on the boundary between local dialects are “*bilingual*” in the sense that they sing both local dialects. Playback experiments have shown the salience of local dialects to males (Pellerin, 1983); for example, males respond differently to playback of own, neighboring, and distant dialects (McGregor, 1983). Another indication of the salience of local dialect variation to males is the example of a polyterritorial male that held territories in neighboring dialects – this male always sang the “*right*” dialect for the territory (McGregor et al., 1997).

**Figure 4:**
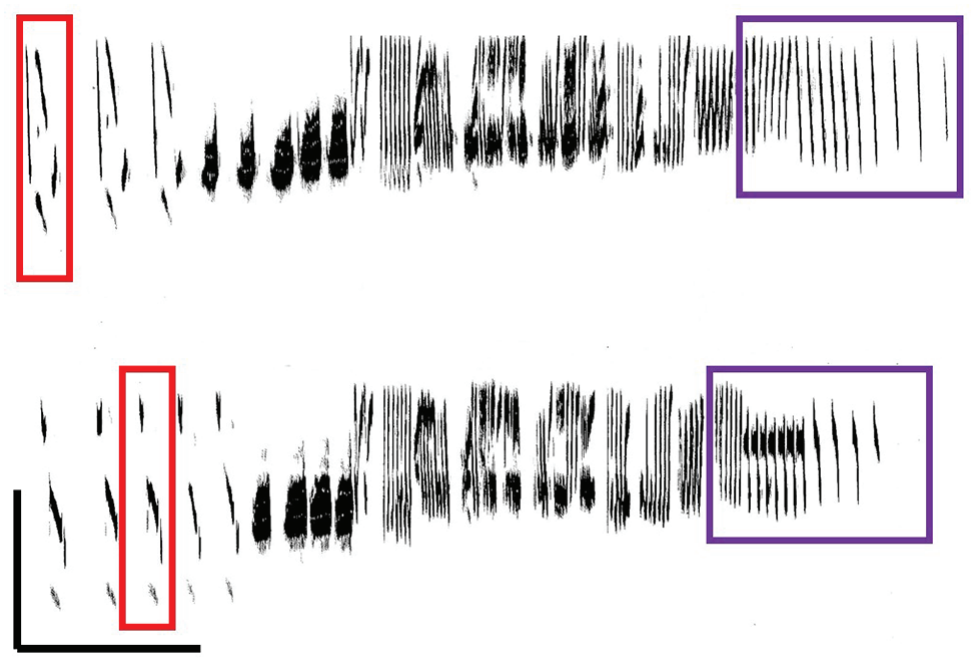
Song of a color-ringed individual male corn bunting in 1987 (upper) and 1990 (lower). Colored boxes show examples of corresponding song elements that have changed between years. Scale bottom left indicates 0–4 kHz (*y*-axis) and 0–0.5 s (*x*-axis). Spectrograms produced by LSI Speech workstation, 16 kHz sampling rate and 160 Hz bandwidth. From original material after McGregor et al. (1997).

A local dialect pattern of song variation has been shown in a number of species (e.g., white-crowned sparrow *Zonotrichia leucophrys*: Marler and Tamura, 1964; village indigo birds *Vidua chalybeata*: Baker and Cunningham, 1985; Payne, 1985). Corn bunting local dialects are comparable to humpback whale song because of a pattern of concerted change in a local dialect, with all males concurrently changing their song to a newer version. The nature of change in corn bunting local dialects from one breeding season to the next is that the detailed note structure of the song changes to varying degrees, but it is still possible to see the relationship to songs from the location recorded several years previously (e.g., McGregor and Thompson, 1988; Holland et al., 1996; McGregor et al., 1997; Figure 4). The surprising aspect of this change over time is that it is a concerted change by all males singing in the population; a study of males individually marked as nestlings showed that no males sang a song from the previous year, even if they had successfully bred then (McGregor et al., 1997). This pattern of concerted change appears comparable to humpback whales in that all males concurrently change their song to a newer version. However, humpback song revolutions are characterized by completely novel arrangements that bear no resemblance to the previous years’ song, whereas the new corn bunting songs show considerable similarity to the previous local dialect – corn bunting song change is evolutionary rather than revolutionary.

## Song Change in Space and Time: Whales and Songbirds

There are clear differences between humpback and corn bunting song variation that may shed some light on the observed differences in cultural transmission and evolution. First, the extent of change in song – wholesale replacement in humpbacks vs. changes in some details of corn bunting local dialects – merits the use of the term song revolution for humpbacks, but not for corn buntings. Second, the geographical scale of change – much more extensive (ocean basin-wide) in humpbacks vs. a few kilometers for corn buntings – this is unremarkable given the differences between species in patterns of movement (corn buntings are sedentary, dispersing a few 100 m from birth to breeding site). However, the associated population size differences (1,000–40,000 individuals in humpbacks vs. 10–100s of males in corn buntings) may have relevance for population genetics. Active singing space, i.e., the number of other individuals in the communication network centered around a singing male, may be a better way to consider the geographical scale of change. Humpback song transmits for tens of kilometers (see Cato, 1991) and could potentially encompass hundreds of individuals, whereas corn bunting song could at best encompass tens of individuals. However, for high-fidelity copying, it is suggested that a whale would need to be within 10 km of the singer to copy higher frequency components of the song that do not transmit as far within the environment; therefore, the size of a network of singers within a breeding ground may be comparable (i.e., tens of individuals in both species). A third major difference is the likely source of the new song variant. In humpbacks, a male from a different and potentially “isolated” humpback population may immigrate into the focal population and initiates a song revolution (Noad et al., 2000). In corn buntings, the source is likely a male from the same population (see below).

There are also some interesting similarities between humpbacks and corn buntings, both in aspects of song acquisition and factors that could affect learning. First, one explanation for the high degree of similarity of songs seen within populations of both species is that the change occurred because a single male produced a new song variant that was then copied, as observed in village indigo birds (Payne, 1985). This raises two questions: how did the new song variant occur and why was this new song copied? The new song variants in corn buntings show relatively limited change, making it more likely that change resulted from a learning or production error at the beginning of the season (McGregor et al., 1997). A similar process may occur in humpback song; recent work using agent-based models of song evolution demonstrated that production errors (*rate* = 1 or 0.1%) facilitated the gradual song evolution observed in the wild (Mcloughlin et al., 2018). Using both novelty bias and production errors in the model mirrored the progressive song evolution observed in the South Pacific (Mcloughlin et al., 2018). However, no scenarios resulted in song revolutions; to produce a revolution in the agent-based model required a song memory to be added (Lamoni, 2018). While learning or production errors are likely causing the gradual evolution of song, innovation cannot be discounted. Understanding whether some males innovate, and to what extent this occurs, or an innovation is copied requires research.

The learning of novel songs seems to be contrary to the arguments for conformity in other species (e.g., Lachlan et al., 2018). The propensity of corn buntings to copy the new song variant may reflect their breeding system. While considered a territorial species, the Hebrides study population is better described as an exploded lek, with polygynous males (one-third total singing males) at the center of the dialect area, monogamous males (also one-third total) surrounding them and unmated males at the periphery. In subsequent years, if males move territories, they do so toward the center and their mating status changes accordingly (Shepherd, 1992; Shepherd et al., 1997). If territory settlement is asynchronous, then the song of the first bird to settle (likely at the dialect center) would be copied (together with any of its learning and/or production errors) by the males settling subsequently in adjacent territories. That is, the current year’s version of the local dialect would spread out from the center of the local dialect area as more males settle. Likewise, the mating system of humpback whales may explain their propensity to copy new song variants including completely novel songs. Humpback whales have a mating system that is polygynous, promiscuous (Clapham, 1996; Clapham and Palsbøll, 1997; Cerchio et al., 2005; Darling et al., 2006), or a lek (including the “floating lek” proposed by Clapham, 1996). In a recent review of song function by Herman (2017), he concluded that humpbacks adhere to a classical lekking system (with a few additions), and the arena is at the scale of a breeding ground (e.g., over 1,000 km^2^). In such a scenario, song changes are unlikely to radiate out from a center akin to corn buntings, as male humpbacks do not hold territories, rather they roam freely (along with females) on the breeding grounds. Song changes are hypothesized to be passed along a chain of singers or to radiate out from a single singer regardless of his position on the breeding grounds. It is unclear which male(s) within the population “*lead*” song changes, if this is a collective process, or if the directional change observed in song units follows an innate template of how songs should evolve (Cerchio et al., 2001). If song evolution is not based on an innate template, who should a male copy? In some songbirds, males copy the song of the most successful breeding male (e.g., Payne, 1985; West and King, 1988). It is unclear how a male humpback whale could assess his reproductive success let alone another male’s. In lieu of a clear indication (e.g., eggs in a nest/fledglings), a feasible method of assessing reproductive “*success*” and a suggestion of whose song to copy would be akin to mate-copying: copy the song of a male who is escorting a female. Males interrupting singers may be prospecting for females (Smith et al., 2008); if a male is unable to displace the current male, he would be a good song model to copy. Whether songs convey fitness information that females use in mate choice is in desperate need of research.

Both humpback whales and corn buntings have suffered extreme population declines through anthropogenic effects, respectively, through whaling (Jackson et al., 2015) and agricultural intensification (in 1970s and 1980s in the UK, central and western Europe; Donald, 1997). While humpbacks are generally recovering worldwide (exception Oceania/western and central South Pacific; Childerhouse et al., 2008; Jackson et al., 2015), UK corn buntings have declined to 10% of their 1970 numbers (Defra, 2018). These different trajectories in population size may help explain the differences in the source of new songs; in recovering humpback populations (such as west or east Australia), immigrating males may bring new songs, whereas new songs originate within declining corn bunting populations.

The effect of declining corn bunting populations seems to be that dialects become less clear (Holland et al., 1996), similar to the less apparent local dialects in less suitable habitats (Osiejuk and Ratynska, 2003). The information on the rate of change of songs in newly established populations is mixed. Songbirds introduced to (and colonizing) areas well outside their natural geographical range may show a slower rate of song change (e.g., chaffinches *Fringilla coelebs* introduced to New Zealand from Sussex, UK; Ince et al., 1980; Jenkins and Baker, 1984) and may retain dialect variation that has been lost from the source population (e.g., yellowhammers *Emberiza citronella*; Pipek et al., 2018). But can also show rapid cultural evolution (e.g., Eurasian tree sparrows *Passer montanus* introduced to the USA; Lang and Barlow, 1997). In the South Pacific, French Polynesia appears to have been recently colonized by humpbacks (Olavarría et al., 2007). French Polynesian song is also perplexing in that song revolutions can fail to take hold (Garland et al., 2011); perhaps this slower rate of song change may be tied to their colonization past. The details of relative isolation of populations and the rate of introgression of males/songs from other populations would seem to be key factors affecting evolutionary and revolutionary change in songs.

## Knowledge Gaps and Future Avenues of Research

Ultimately, the aim of this comparative approach is to understand population-wide processes in the natural environment that could apply to any species of animal. In whales and songbirds, a few specific data gaps are clear; filling these, while extremely challenging to say the least, is what we should be striving for. Different species may be more amenable to address specific questions than others. Long-term datasets are key to answering questions in cultural evolution. These can take a researcher’s lifetime to build and to address processes and mechanisms will require information at an individual level on genotype, song samples (through time), reproductive success, and movements (to name a few). The following list of questions in key topics needs to be addressed:

- Innovation. Do some or all males innovate? To what extent does innovation occur? Are all innovations copied? How do males choose which male (or innovation!) to copy?
- Song and mating. Do songs (or parts of songs) convey fitness information? And if so, what aspects do females use in mate choice? Is novelty part of the answer?
- Source of variation. Is song evolution primarily caused by production errors?
- Anthropogenic influences. Are human-induced changes in population size and distribution critical factors driving revolution, innovation, and local dialects?

To test these questions, meticulous individual-level data are needed for some (e.g., song, or song parts, conveying fitness), while other broader-scale questions (e.g., production errors) can be addressed by population surveillance. For example, the study of cultural evolution of savannah sparrow song over three decades showed a change in some song elements but not others, and variants of some parts of the song were associated with reproductive success (Williams et al., 2013). In terms of other tractable systems, testing such questions using corn buntings (or other applicable species, e.g., village indigo birds) would greatly enhance our wider understanding of song evolution. There is undoubtedly a strong genetic basis to singing and song, where the genetic influence ends and cultural processes begin is where things get interesting. Unfortunately, no songbird or any other animal species to date have exhibited the rapid and repeated population-wide replacement of a cultural phenotype as observed in humpback song revolutions. However, the recent report that a variant of white-throated sparrow song spread across Canada in a west to east direction replacing the existing song is similar in the extent of change but much slower in that the change took some decades (Otter et al., 2020).

There is one final piece to the cultural puzzle we have not considered here: human culture. Cultural evolution, language evolution, horizontal transmission of fads, and the like all provide a rich expansion for the comparative approach we have taken here (Smith and Kirby, 2008; Whiten et al., 2017). The spread of pop songs or fashion through a population bears a striking resemblance to the spread of song revolutions. Markers of group identity shown through the rapid cultural change in slang words within a group are also present in shared songbird dialects. With the maturing of multiple long-term datasets, the next decade is poised to provide answers to some of these important questions in cultural evolution.

## Author Contributions

EG and PM conceived of the study, drafted the manuscript, critically revised it, and provided approval for publication of the final version.

### Conflict of Interest

The authors declare that the research was conducted in the absence of any commercial or financial relationships that could be construed as a potential conflict of interest.
